# Association of pulse pressure with fasting and 2-h plasma glucose in subjects with different degrees of glucose tolerance

**DOI:** 10.1186/1758-5996-7-S1-A32

**Published:** 2015-11-11

**Authors:** Letícia Maria Tedesco Silva, Vanessa Piccoli, Barbara Limberger Nedel, Tássia Cividanes Pazinato, Leonardo de Andrade Mesquita, Luciana Pavan Antoniolli, Rodrigo Soares de Souza Marques, Fernando Gerchman

**Affiliations:** 1 Hospital de Clínicas de Porto Alegre, Porto Alegre, Brazil

## Background

Increased pulse pressure (PP) is a strong predictor of cardiovascular mortality and it has been associated with an increased risk of developing diabetes and its microvascular complications. However, the mechanism underlying the association between PP and abnormalities of glucose metabolism is unclear.

## Objective

To study how PP obtained by 24h ambulatory blood pressure monitoring (ABPM) is related to fasting and 2-h plasma glucose in subjects with different degrees of glucose tolerance.

## Materials and methods

In a cross-sectional study, 128 subjects (53.1±12.3 y, females 72%) were submitted to a 75-g OGTT (measurement of glucose and insulin) and divided according to glucose tolerance status (normal glucose tolerance [NGT; n=38], prediabetes [PDM; n=53] and diabetes [DM; n=37]). 24-h ABPM was performed. Mean 24h PP was calculated as the difference between mean systolic and diastolic blood pressure (BP), obtained through 24-h ABPM. Fasting C-peptide and A1c were collected. Body size (BMI) and central obesity (waist circumference) were assessed. Insulin sensitivity index (ISI Stumvoll), insulin resistance (HOMA-IR) and β-cell function (insulinogenic index; ΔIns30′-0′/ΔGli30′-0′) were estimated. A two-sided P value < 0.05 was considered significant.

## Results

By ABPM, 24-hour PP progressively increased from NGT to DM (mean±SD; NGT 45.9±8.5 vs PDM 51.9±10.4 vs DM 57.8±11.1 mmHg; P<0.001). 24-hour PP was positively related to age (r=0.316; P<0.001), waist circumference (r=0.263; P=0.003), BMI (r=0.35; P<0.001), A1C (r=0.438; P<0.001), 2-h glucose level (r=0.424; P<0.001), C-peptide (r=0.286; P=0.001), HOMA-IR (r=0.155; P=0.085) and it was inversely related to ISI Stumvoll (r=-0.474; P <0.001) and to insulinogenic index (r=-0.184; P=0.048). While adjusting for age and waist circumference, pulse pressure was independently associated with 2 hour plasma glucose (R=0.287; P=0.002) and A1c (R=0.241; P=0.010).

## Conclusion

According to our data, pulse pressure obtained by 24-h ABPM increases with decreasing of glucose tolerance. It is also an independent predictor of higher 2 hour plasma glucose and A1c levels.

